# From Policy to Practice: Evaluating the Impact of Alzheimer’s State Plans Across the U.S.

**DOI:** 10.1093/geroni/igaf122.3392

**Published:** 2025-12-31

**Authors:** KoriAnne Moslander, Christopher Kelly, Julie Blaskewicz Boron

**Affiliations:** University of Nebraska Omaha, Omaha, Nebraska, United States; University of Nebraska Omaha, Omaha, Nebraska, United States; University of Nebraska Omaha, Omaha, Nebraska, United States

## Abstract

The National Alzheimer’s Project Act was signed into law on January 4, 2011, establishing the Advisory Council on Alzheimer’s Research, Care, and Services, creating a national plan, and supporting states serving this population. This study utilized a policy analysis framework to examine the implementation of Silver Alert, early detection programs, and dementia training requirements across all 50 states. Further analysis of these policies identified the success rates of the proposed policies compared across each state. It exposed the gaps present in serving the residents of each state living with Alzheimer’s disease and other dementias. Results revealed that 64% of states, along with the District of Columbia and Puerto Rico, have implemented a Silver Alerting program or similar, require a certain level of dementia training, and provide early detection programs and campaigns. The leading diagnostic test for early detection of dementia is biomarker testing. Legislation to ensure coverage for this test has passed in 16 states. Illinois was the first state to enact this legislation in August of 2024. Washington state has the greatest number of hours required for dementia training for direct care workers, with 12 hours pertinent to dementia care. California requires fewer but mandates two hours of dementia training within the first 40 hours of employment and five hours of dementia-specific in-service training annually. Florida, California, and Utah lead with the most success stories from their Silver Alert programs. Further discussion will examine the substantial progress made in these state’s Alzheimer’s state plans.

